# 2-(1-Phenyl-1*H*-benzimidazol-2-yl)phenol

**DOI:** 10.1107/S1600536812049859

**Published:** 2012-12-08

**Authors:** A. Thiruvalluvar, S. Rosepriya, K. Jayamoorthy, J. Jayabharathi, Sema Öztürk Yildirim, R. J. Butcher

**Affiliations:** a Postgraduate Research Department of Physics, Rajah Serfoji Government College (Autonomous), Thanjavur 613 005, Tamilnadu, India; b Department of Chemistry, Annamalai University, Annamalai Nagar 608 002, Tamilnadu, India; c Department of Chemistry, Howard University, 525 College Street NW, Washington, DC 20059, USA; d Department of Physics, Faculty of Sciences, Erciyes University, 38039 Kayseri, Turkey

## Abstract

In the title mol­ecule, C_19_H_14_N_2_O, the benzimidazole unit is close to being planar [maximum deviation = 0.0253 (11) Å] and forms dihedral angles of 68.98 (6) and 20.38 (7)° with the adjacent phenyl and benzene rings; the dihedral angle between the latter two planes is 64.30 (7)°. An intra­molecular O—H⋯N hydrogen bond generates an *S*(6) ring motif. In the crystal, mol­ecules are linked by C—H⋯N and C—H⋯O hydrogen bonds, and consolidated into a three-dimensional architecture by π–π stacking inter­actions, with a centroid–centroid distance of 3.8428 (12) Å.

## Related literature

For the range of pharmacological activities and toxicological properties of benzimidazole derivatives, see: Spasov *et al.* (1999 ▶). For closely related crystal structures, see: Jayamoorthy *et al.* (2012 ▶); Rosepriya *et al.* (2012 ▶). For hydrogen-bond motifs, see: Bernstein *et al.* (1995 ▶).
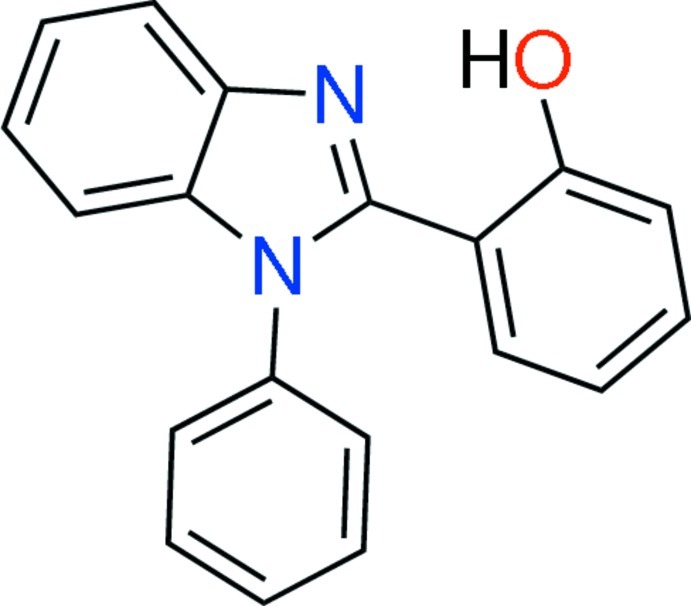


## Experimental

### 

#### Crystal data

C_19_H_14_N_2_O
*M* *_r_* = 286.32
Triclinic, 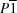

*a* = 8.1941 (6) Å
*b* = 9.5983 (14) Å
*c* = 10.3193 (18) Å
α = 64.637 (16)°
β = 80.356 (10)°
γ = 83.610 (9)°
*V* = 722.3 (2) Å^3^
*Z* = 2
Cu *K*α radiation
μ = 0.66 mm^−1^
*T* = 123 K
0.76 × 0.46 × 0.32 mm

#### Data collection

Agilent Xcalibur Ruby Gemini diffractometer
Absorption correction: analytical [*CrysAlis PRO* (Agilent, 2012 ▶), using a multi-faceted crystal model (Clark & Reid, 1995 ▶)] *T*_min_ = 0.731, *T*_max_ = 0.811
4335 measured reflections
2826 independent reflections
2420 reflections with *I* > 2σ(*I*)
*R*_int_ = 0.076

#### Refinement

*R*[*F* ^2^ > 2σ(*F* ^2^)] = 0.059
*wR*(*F* ^2^) = 0.171
*S* = 1.04
2826 reflections
203 parameters
H atoms treated by a mixture of independent and constrained refinement
Δρ_max_ = 0.32 e Å^−3^
Δρ_min_ = −0.31 e Å^−3^

### 

Data collection: *CrysAlis PRO* (Agilent, 2012 ▶); cell refinement: *CrysAlis PRO*; data reduction: *CrysAlis PRO*; program(s) used to solve structure: *SHELXS86* (Sheldrick, 2008 ▶); program(s) used to refine structure: *SHELXL97* (Sheldrick, 2008 ▶); molecular graphics: *ORTEP-3 for Windows* (Farrugia, 2012 ▶) and *PLATON* (Spek, 2009 ▶); software used to prepare material for publication: *PLATON*.

## Supplementary Material

Crystal structure: contains datablock(s) global, I. DOI: 10.1107/S1600536812049859/tk5179sup1.cif

Structure factors: contains datablock(s) I. DOI: 10.1107/S1600536812049859/tk5179Isup2.hkl

Supplementary material file. DOI: 10.1107/S1600536812049859/tk5179Isup3.cdx

Supplementary material file. DOI: 10.1107/S1600536812049859/tk5179Isup4.cml

Additional supplementary materials:  crystallographic information; 3D view; checkCIF report

## Figures and Tables

**Table 1:** Hydrogen-bond geometry (Å, °)

*D*—H⋯*A*	*D*—H	H⋯*A*	*D*⋯*A*	*D*—H⋯*A*
O26—H26⋯N3	0.97 (3)	1.70 (3)	2.583 (2)	150 (3)
C14—H14⋯N3^i^	0.95	2.60	3.456 (3)	151
C16—H16⋯O26^ii^	0.95	2.49	3.388 (2)	157

Symmetry codes: (i) 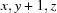; (ii) 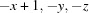.

**Fig. 1.**
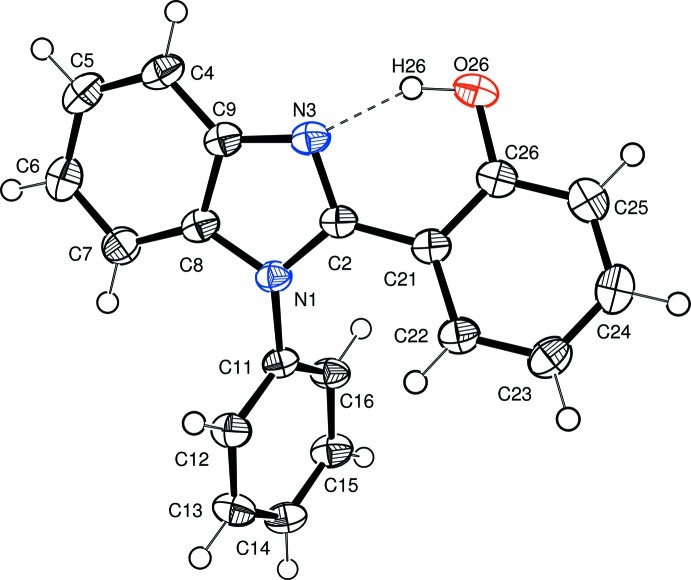
The molecular structure of the title compound, with displacement ellipsoids drawn at the 50% probability level. H atoms are shown as small spheres of arbitrary radius. The dashed line indicates the intramolecular O—H···N hydrogen bond.

**Fig. 2.**
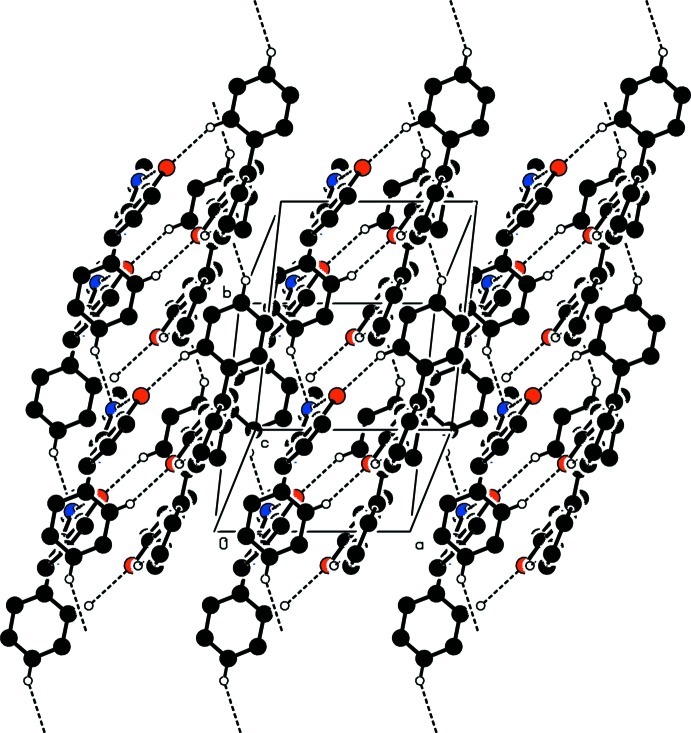
The packing of the title compound, viewed approximately down the c axis. Dashed lines indicate hydrogen bonds. H atoms not involved in hydrogen bonding have been omitted.

**Fig. 3.**
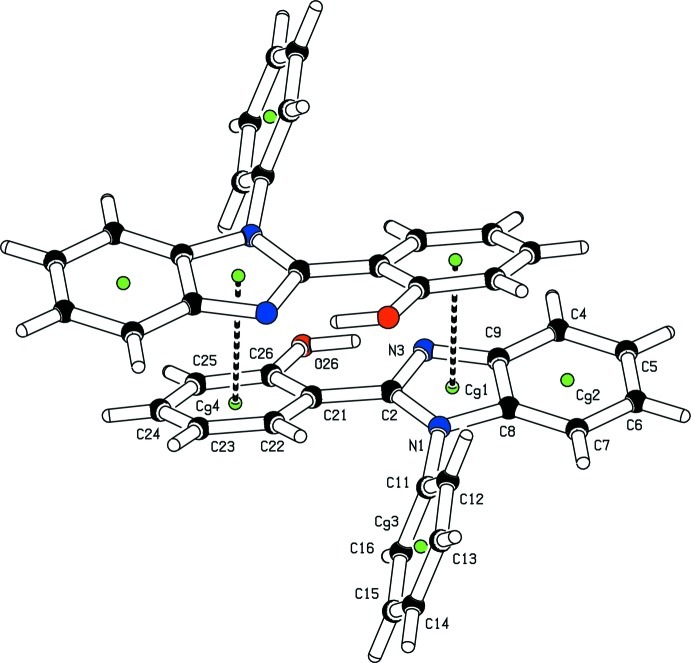
Part of the crystal structure of compound, showing the formation of π—π stacking interactions. Symmetry code: 2 - x, - y, - z

## supplementary crystallographic information

### Comment

Spasov *et al.* (1999) have reviewed the wide range of pharmacological activities and toxicological properties of benzimidazole derivatives. Since our research group is working in organic light emitting devices, we are interested to use the title compound as ligand for synthesizing Ir^III^ complexes. Further, we are interested to use the title compound as a ligand to study excited state intramolecular proton transfer (ESIPT) processes. Jayamoorthy *et al.* (2012) and Rosepriya *et al.* (2012) have reported closely related crystal structures of benzimidazole derivatives.

In the title molecule, C_19_H_14_N_2_O (Fig. 1), the benzimidazole unit is almost planar [maximum deviation = 0.0253 (11) Å for C2]. The dihedral angles between the planes of the benzimidazole and the phenyl ring at N1 and the benzene ring at C2 are 68.98 (6) and 20.38 (7)°, respectively. The dihedral angle between the planes of the adjacent phenyl and benzene rings is 64.30 (7)°. The molecular conformation is stabilized by an intramolecular O26—H26···N3 hydrogen bond, which generates an *S*(6) ring motif (Bernstein *et al.*, 1995). In the crystal (Fig. 2), molecules are linked by C14—H14···N3 and C16—H16···O26 hydrogen bonds (Table 1). Further, π—π stacking interactions between symmetry-related imidazole and benzene rings [*Cg*1—*Cg*4^iii^ = *Cg*4—*Cg*1^iii^ = 3.8428 (12) Å, symmetry code (iii): 2 - *x*, - *y*, - *z* where *Cg*1 is the centroid of the imidazole ring (N1/C2/N3/C9/C8) and *Cg*4 is the centroid of the benzene ring defined by atoms C21—C26, respectively] (Fig. 3) are noted.

### Experimental

To *N*-phenyl-*o*-phenylenediamine (3.128 g, 17 mmol) in ethanol (10 ml), 2-hydroxybenzaldehyde (1.8 ml, 17 mmol) and ammonium acetate (4 g) were added over about 1 h while maintaining the temperature at 353 K. The reaction mixture was refluxed for the appropriate time and the completion of reaction was monitored by TLC. The reaction mixture extracted with dichloromethane. The solid that separated was purified by column chromatography using petroleum ether (60–80 °C) as the eluent. Yield: 2.43 g (50%). The title compound was dissolved in petroleum ether and chloroform (9:1) mixture and allowed to slow evaporate for two days to obtain crystals suitable for X-ray diffraction studies.

### Refinement

The O-bound H atom was located in a difference Fourier map and refined freely; O26—H26 = 0.97 (3) Å. The remaining H atoms were positioned geometrically and allowed to ride on their parent atoms, with C—H = 0.95 Å, and with *U*_iso_(H) = *1.2U*_eq_(C).

### Crystal data

**Table:**

C_19_H_14_N_2_O	*Z* = 2
*M**_r_* = 286.32	*F*(000) = 300
Triclinic, *P*1	*D*_x_ = 1.316 Mg m^−^^3^
Hall symbol: -P 1	Melting point: 387 K
*a* = 8.1941 (6) Å	Cu *K*α radiation, λ = 1.54184 Å
*b* = 9.5983 (14) Å	Cell parameters from 772 reflections
*c* = 10.3193 (18) Å	θ = 4.8–75.4°
α = 64.637 (16)°	µ = 0.66 mm^−^^1^
β = 80.356 (10)°	*T* = 123 K
γ = 83.610 (9)°	Block, colourless
*V* = 722.3 (2) Å^3^	0.76 × 0.46 × 0.32 mm

### Data collection

**Table:**

Agilent Xcalibur Ruby Gemini diffractometer	2826 independent reflections
Radiation source: Enhance (Cu) X-ray Source	2420 reflections with *I* > 2σ(*I*)
Graphite monochromator	*R*_int_ = 0.076
Detector resolution: 10.5081 pixels mm^-1^	θ_max_ = 75.8°, θ_min_ = 5.5°
ω scans	*h* = −10→10
Absorption correction: analytical [*CrysAlis PRO* (Agilent, 2012), using a multi-faceted crystal model (Clark & Reid, 1995)]	*k* = −9→12
*T*_min_ = 0.731, *T*_max_ = 0.811	*l* = −12→12
4335 measured reflections	

### Refinement

**Table:**

Refinement on *F*^2^	Primary atom site location: structure-invariant direct methods
Least-squares matrix: full	Secondary atom site location: difference Fourier map
*R*[*F*^2^ > 2σ(*F*^2^)] = 0.059	Hydrogen site location: inferred from neighbouring sites
*wR*(*F*^2^) = 0.171	H atoms treated by a mixture of independent and constrained refinement
*S* = 1.04	*w* = 1/[σ^2^(*F*_o_^2^) + (0.1182*P*)^2^ + 0.1646*P*] where *P* = (*F*_o_^2^ + 2*F*_c_^2^)/3
2826 reflections	(Δ/σ)_max_ = 0.001
203 parameters	Δρ_max_ = 0.32 e Å^−^^3^
0 restraints	Δρ_min_ = −0.31 e Å^−^^3^

### Special details

**Table:**

Geometry. Bond distances, angles *etc*. have been calculated using the rounded fractional coordinates. All su's are estimated from the variances of the (full) variance-covariance matrix. The cell e.s.d.'s are taken into account in the estimation of distances, angles and torsion angles
Refinement. Refinement of *F*^2^ against ALL reflections. The weighted *R*-factor *wR* and goodness of fit *S* are based on *F*^2^, conventional *R*-factors *R* are based on *F*, with *F* set to zero for negative *F*^2^. The threshold expression of *F*^2^ > 2σ(*F*^2^) is used only for calculating *R*-factors(gt) *etc*. and is not relevant to the choice of reflections for refinement. *R*-factors based on *F*^2^ are statistically about twice as large as those based on *F*, and *R*- factors based on ALL data will be even larger.

### Fractional atomic coordinates and isotropic or equivalent isotropic displacement parameters (Å^2^)

**Table:**

	*x*	*y*	*z*	*U*_iso_*/*U*_eq_	
O26	0.61570 (15)	−0.11111 (15)	−0.08352 (15)	0.0336 (4)	
N1	0.78361 (16)	0.03924 (16)	0.19436 (15)	0.0249 (4)	
N3	0.70983 (17)	−0.15812 (16)	0.15919 (16)	0.0262 (4)	
C2	0.75812 (18)	−0.01378 (18)	0.09515 (18)	0.0238 (4)	
C4	0.6537 (2)	−0.3401 (2)	0.4242 (2)	0.0331 (5)	
C5	0.6508 (2)	−0.3488 (2)	0.5613 (2)	0.0384 (5)	
C6	0.6940 (3)	−0.2238 (2)	0.5834 (2)	0.0386 (6)	
C7	0.7442 (2)	−0.0876 (2)	0.4680 (2)	0.0340 (5)	
C8	0.7465 (2)	−0.07982 (18)	0.33084 (19)	0.0272 (5)	
C9	0.70108 (19)	−0.20226 (19)	0.30605 (19)	0.0269 (5)	
C11	0.8035 (2)	0.19445 (18)	0.17495 (18)	0.0250 (5)	
C12	0.9424 (2)	0.2276 (2)	0.2142 (2)	0.0301 (5)	
C13	0.9554 (2)	0.3752 (2)	0.2042 (2)	0.0344 (5)	
C14	0.8320 (2)	0.4876 (2)	0.1542 (2)	0.0331 (5)	
C15	0.6936 (2)	0.4524 (2)	0.1156 (2)	0.0345 (5)	
C16	0.6775 (2)	0.30484 (19)	0.1263 (2)	0.0306 (5)	
C21	0.77729 (18)	0.07290 (18)	−0.06192 (18)	0.0245 (5)	
C22	0.8703 (2)	0.2056 (2)	−0.13605 (19)	0.0286 (5)	
C23	0.8821 (2)	0.2863 (2)	−0.2849 (2)	0.0343 (5)	
C24	0.7977 (2)	0.2376 (2)	−0.3632 (2)	0.0359 (5)	
C25	0.7070 (2)	0.1065 (2)	−0.2933 (2)	0.0330 (5)	
C26	0.69972 (19)	0.0214 (2)	−0.14465 (19)	0.0272 (5)	
H4	0.62439	−0.42523	0.41012	0.0397*	
H5	0.61885	−0.44136	0.64270	0.0460*	
H6	0.68856	−0.23287	0.67934	0.0463*	
H7	0.77567	−0.00349	0.48246	0.0408*	
H12	1.02792	0.15069	0.24756	0.0361*	
H13	1.04993	0.39890	0.23189	0.0413*	
H14	0.84218	0.58863	0.14649	0.0397*	
H15	0.60863	0.52966	0.08129	0.0414*	
H16	0.58178	0.28037	0.10080	0.0367*	
H22	0.92633	0.24072	−0.08287	0.0343*	
H23	0.94752	0.37448	−0.33327	0.0411*	
H24	0.80249	0.29466	−0.46494	0.0431*	
H25	0.64902	0.07421	−0.34740	0.0395*	
H26	0.629 (4)	−0.158 (3)	0.018 (3)	0.057 (7)*	

### Atomic displacement parameters (Å^2^)

**Table:**

	*U*^11^	*U*^22^	*U*^33^	*U*^12^	*U*^13^	*U*^23^
O26	0.0296 (6)	0.0337 (7)	0.0436 (8)	−0.0070 (5)	−0.0042 (5)	−0.0208 (6)
N1	0.0230 (7)	0.0213 (7)	0.0301 (7)	−0.0004 (5)	−0.0039 (5)	−0.0105 (6)
N3	0.0218 (6)	0.0218 (7)	0.0342 (8)	−0.0001 (5)	−0.0030 (5)	−0.0113 (6)
C2	0.0175 (7)	0.0225 (7)	0.0316 (8)	0.0013 (5)	−0.0026 (6)	−0.0123 (6)
C4	0.0269 (8)	0.0230 (8)	0.0422 (10)	−0.0008 (6)	−0.0021 (7)	−0.0078 (7)
C5	0.0349 (9)	0.0295 (9)	0.0374 (10)	−0.0012 (7)	0.0001 (8)	−0.0032 (8)
C6	0.0390 (10)	0.0397 (10)	0.0310 (9)	0.0025 (8)	−0.0034 (8)	−0.0106 (8)
C7	0.0360 (9)	0.0297 (9)	0.0360 (9)	0.0011 (7)	−0.0065 (7)	−0.0135 (8)
C8	0.0222 (7)	0.0231 (8)	0.0335 (9)	0.0006 (6)	−0.0020 (6)	−0.0103 (7)
C9	0.0209 (7)	0.0236 (8)	0.0342 (9)	0.0019 (6)	−0.0034 (6)	−0.0111 (7)
C11	0.0252 (8)	0.0201 (8)	0.0287 (8)	−0.0022 (6)	−0.0007 (6)	−0.0100 (6)
C12	0.0244 (8)	0.0262 (8)	0.0401 (10)	0.0016 (6)	−0.0068 (7)	−0.0140 (7)
C13	0.0265 (8)	0.0341 (10)	0.0474 (10)	−0.0045 (7)	−0.0055 (7)	−0.0206 (8)
C14	0.0331 (9)	0.0237 (8)	0.0422 (10)	−0.0041 (7)	0.0008 (7)	−0.0149 (7)
C15	0.0289 (9)	0.0243 (8)	0.0481 (11)	0.0034 (6)	−0.0053 (7)	−0.0141 (8)
C16	0.0234 (8)	0.0251 (8)	0.0433 (10)	−0.0007 (6)	−0.0068 (7)	−0.0135 (7)
C21	0.0170 (7)	0.0243 (8)	0.0322 (9)	0.0023 (6)	−0.0018 (6)	−0.0130 (7)
C22	0.0208 (7)	0.0293 (9)	0.0347 (9)	−0.0009 (6)	−0.0013 (6)	−0.0134 (7)
C23	0.0285 (9)	0.0318 (9)	0.0357 (10)	−0.0009 (7)	0.0019 (7)	−0.0100 (8)
C24	0.0316 (9)	0.0406 (10)	0.0300 (9)	0.0077 (7)	−0.0027 (7)	−0.0122 (8)
C25	0.0259 (8)	0.0399 (10)	0.0368 (10)	0.0059 (7)	−0.0054 (7)	−0.0208 (8)
C26	0.0189 (7)	0.0295 (8)	0.0352 (9)	0.0029 (6)	−0.0018 (6)	−0.0170 (7)

### Geometric parameters (Å, º)

**Table:**

O26—C26	1.361 (2)	C21—C26	1.411 (3)
O26—H26	0.97 (3)	C21—C22	1.405 (3)
N1—C2	1.376 (2)	C22—C23	1.384 (3)
N1—C8	1.392 (2)	C23—C24	1.390 (3)
N1—C11	1.440 (2)	C24—C25	1.381 (3)
N3—C9	1.379 (2)	C25—C26	1.389 (3)
N3—C2	1.328 (2)	C4—H4	0.9500
C2—C21	1.460 (2)	C5—H5	0.9500
C4—C9	1.401 (3)	C6—H6	0.9500
C4—C5	1.377 (3)	C7—H7	0.9500
C5—C6	1.405 (3)	C12—H12	0.9500
C6—C7	1.385 (3)	C13—H13	0.9500
C7—C8	1.382 (3)	C14—H14	0.9500
C8—C9	1.404 (3)	C15—H15	0.9500
C11—C12	1.381 (2)	C16—H16	0.9500
C11—C16	1.384 (3)	C22—H22	0.9500
C12—C13	1.391 (3)	C23—H23	0.9500
C13—C14	1.381 (3)	C24—H24	0.9500
C14—C15	1.382 (2)	C25—H25	0.9500
C15—C16	1.392 (3)		
			
C26—O26—H26	106.6 (19)	C23—C24—C25	120.11 (17)
C2—N1—C8	106.88 (15)	C24—C25—C26	120.62 (17)
C8—N1—C11	121.93 (15)	O26—C26—C25	117.70 (16)
C2—N1—C11	129.56 (15)	C21—C26—C25	120.26 (17)
C2—N3—C9	106.46 (15)	O26—C26—C21	122.04 (16)
N1—C2—C21	126.44 (16)	C5—C4—H4	121.00
N3—C2—C21	121.90 (16)	C9—C4—H4	121.00
N1—C2—N3	111.66 (15)	C4—C5—H5	119.00
C5—C4—C9	118.13 (18)	C6—C5—H5	119.00
C4—C5—C6	121.46 (18)	C5—C6—H6	119.00
C5—C6—C7	121.27 (18)	C7—C6—H6	119.00
C6—C7—C8	116.87 (18)	C6—C7—H7	122.00
N1—C8—C7	131.52 (18)	C8—C7—H7	122.00
N1—C8—C9	105.57 (15)	C11—C12—H12	120.00
C7—C8—C9	122.88 (17)	C13—C12—H12	120.00
N3—C9—C8	109.43 (16)	C12—C13—H13	120.00
C4—C9—C8	119.38 (17)	C14—C13—H13	120.00
N3—C9—C4	131.18 (18)	C13—C14—H14	120.00
N1—C11—C16	119.08 (15)	C15—C14—H14	120.00
C12—C11—C16	121.35 (18)	C14—C15—H15	120.00
N1—C11—C12	119.43 (16)	C16—C15—H15	120.00
C11—C12—C13	119.07 (17)	C11—C16—H16	121.00
C12—C13—C14	120.50 (17)	C15—C16—H16	121.00
C13—C14—C15	119.69 (19)	C21—C22—H22	119.00
C14—C15—C16	120.66 (17)	C23—C22—H22	119.00
C11—C16—C15	118.73 (16)	C22—C23—H23	120.00
C2—C21—C26	118.85 (16)	C24—C23—H23	120.00
C22—C21—C26	117.79 (16)	C23—C24—H24	120.00
C2—C21—C22	123.35 (16)	C25—C24—H24	120.00
C21—C22—C23	121.43 (17)	C24—C25—H25	120.00
C22—C23—C24	119.65 (18)	C26—C25—H25	120.00
			
C8—N1—C2—N3	−0.83 (19)	C6—C7—C8—N1	177.26 (19)
C8—N1—C2—C21	178.31 (15)	C6—C7—C8—C9	−0.1 (3)
C11—N1—C2—N3	−166.19 (16)	N1—C8—C9—N3	−0.26 (19)
C11—N1—C2—C21	13.0 (3)	N1—C8—C9—C4	−179.06 (15)
C2—N1—C8—C7	−177.06 (18)	C7—C8—C9—N3	177.69 (16)
C2—N1—C8—C9	0.64 (18)	C7—C8—C9—C4	−1.1 (3)
C11—N1—C8—C7	−10.3 (3)	N1—C11—C12—C13	−175.74 (16)
C11—N1—C8—C9	167.36 (15)	C16—C11—C12—C13	−0.1 (3)
C2—N1—C11—C12	−124.86 (19)	N1—C11—C16—C15	176.42 (16)
C2—N1—C11—C16	59.4 (2)	C12—C11—C16—C15	0.8 (3)
C8—N1—C11—C12	71.7 (2)	C11—C12—C13—C14	−0.7 (3)
C8—N1—C11—C16	−104.04 (19)	C12—C13—C14—C15	0.8 (3)
C9—N3—C2—N1	0.66 (19)	C13—C14—C15—C16	−0.2 (3)
C9—N3—C2—C21	−178.53 (15)	C14—C15—C16—C11	−0.6 (3)
C2—N3—C9—C4	178.37 (18)	C2—C21—C22—C23	−178.51 (17)
C2—N3—C9—C8	−0.23 (19)	C26—C21—C22—C23	1.8 (3)
N1—C2—C21—C22	19.6 (3)	C2—C21—C26—O26	−3.8 (3)
N1—C2—C21—C26	−160.78 (16)	C2—C21—C26—C25	176.02 (16)
N3—C2—C21—C22	−161.38 (17)	C22—C21—C26—O26	175.86 (16)
N3—C2—C21—C26	18.3 (2)	C22—C21—C26—C25	−4.3 (3)
C9—C4—C5—C6	−0.1 (3)	C21—C22—C23—C24	1.4 (3)
C5—C4—C9—N3	−177.34 (17)	C22—C23—C24—C25	−2.1 (3)
C5—C4—C9—C8	1.2 (3)	C23—C24—C25—C26	−0.4 (3)
C4—C5—C6—C7	−1.2 (3)	C24—C25—C26—O26	−176.50 (17)
C5—C6—C7—C8	1.2 (3)	C24—C25—C26—C21	3.7 (3)

### Hydrogen-bond geometry (Å, º)

**Table:**

*D*—H···*A*	*D*—H	H···*A*	*D*···*A*	*D*—H···*A*
O26—H26···N3	0.97 (3)	1.70 (3)	2.583 (2)	150 (3)
C14—H14···N3^i^	0.95	2.60	3.456 (3)	151
C16—H16···O26^ii^	0.95	2.49	3.388 (2)	157

Symmetry codes: (i) *x*, *y*+1, *z*; (ii) −*x*+1, −*y*, −*z*.
